# A case of lichenoid photosensitive eczema in a HIV seropositive patient

**DOI:** 10.1186/1471-2334-14-S3-P54

**Published:** 2014-05-27

**Authors:** R Gayathri, AK Bubna, M Krishnakanth, S Adikrishnan, S Murugan, V Mahalakshmi, S Anandan, R Sudha

**Affiliations:** 1 Department of Dermatology, Venereology and Leprosy, Sri Ramachandra Medical College, Chennai, India

## Background

Cutaneous changes in HIV have protean manifestations. We hereby present a case of a lichenoid photosensitive eczema in a HIV positive patient.

## Case report

A 32 year old female presented with burning and itching sensation with hyperpigmentation over the sun exposed areas- face, neck, back and arms. No history of any blisters over skin or hypertrichosis. History of weight loss of more than 6 kgs was documented in a month. Patient’s husband was a driver with history of multiple unprotected sexual exposures with CSW. No history of any oral or genital ulcers, artharlgia, diarrhoeal episodes, evening rise of temperature and cough with expectoration.

O/E: Erythematous and edematous plaques present over sun exposed areas. Hyperpigmention with lichenoid hue + over malar region, neck folds ,back and arms Multiple excoriation marks, hyperpigmented papules and plaques present over bilateral arms, legs, and ante cubital fossa.Oral cavity, genital mucosas were normal. No generalised lymphadenopathy. Routine laboratory investigations were normal. Mantoux test, RPR, Chest X- ray, USG abdomen pelvis was normal. Skin biopsy was suggestive of interface dermatitis. HIV (1 and 2) ELISA was reactive which was confirmed by Western blot. The CD4 count was 610/cumm.

## Discussion

Lichenoid photosensitive eczema is known to manifest in the terminal stage of the HIV infection in individuals with a low CD4 count. Our patient is a newly diagnosed case of HIV with a relatively high CD4 count manifesting with photosensitive eczema. This case is presented for its rarity.

